# Neuromodulation using Bioelectronics-enabled therapies to combat insulin resistance and its comorbidities

**DOI:** 10.1007/s40200-026-01876-w

**Published:** 2026-02-13

**Authors:** Yosra Magdi Mekki, Jeongeun Park, Zeinah Alzaareer, Zaeem Hussain, Ayesha Gulzar, Abdelnaser Elzouki, Amparo Güemes, Susu M. Zughaier

**Affiliations:** 1 Department of Basic Medical Sciences, College of Medicine, QU Health, Qatar University, Doha, Qatar; 2 Department of Engineering, Electrical Engineering Division, University of Cambridge, 9 JJ Thomson Ave, Cambridge, CB3 0FA UK; 3 Department of Internal Medicine, Hamad Medical Corporation, Doha, Qatar; 4 Weill Cornell College of Medicine, New York, NY USA; 5 Weill Cornell College of Medicine, Doha, Qatar

**Keywords:** Neuromodulation, Bioelectronic medicine, Vagal nerve stimulation, Insulin resistance

## Abstract

**Introduction:**

Insulin resistance is a complex metabolic disorder that involves multiple molecular pathways to disrupt insulin signaling and is associated with systemic complications.

**Methodology:**

We searched through the literature using databases such as PubMed and selected relevant published papers related to the topic of this review.

**Results:**

We synthesized this review based on the relevant literature and found that neuromodulation can target neuroendocrine systems responsible for metabolic homeostasis to manage insulin resistance. This involves integrating neural circuit modulation into biological systems. We also found that neuromodulation, particularly vagus nerve stimulation, can reduce inflammation and combat insulin resistance and its associated comorbidities. However, with advancements in closed-loop systems and artificial intelligence, challenges persist in maximizing therapeutic effects, minimizing adverse events, and balancing ethical considerations.

**Conclusion:**

Neuromodulation offers novel treatment options for metabolic disorders. An enhanced understanding of neuro-metabolic reflex in insulin resistance is needed to develop more effective and safer neuromodulation therapy.

## Introduction

### Insulin resistance: A comprehensive metabolic challenge

The complexity of insulin resistance (IR) is emphasized due to its multifactorial pathophysiology, which involves several physiological processes and molecular pathways. Evidence suggests that physiological insulin signaling pathways may be disrupted by genetic factors such as mutations of receptors and proteins involved in the signaling process [1, 2]. Moreover, disturbances in organ crosstalk that maintain metabolic homeostasis and insulin sensitivity, along with hormonal imbalances, can potentially cause IR. Therefore, the cellular insulin response is impaired, leading to an imbalanced glucose metabolism. This section provides an overview of these mechanisms and the associated comorbidities of IR.

### Molecular mechanisms of insulin resistance

**Fig. 1:**
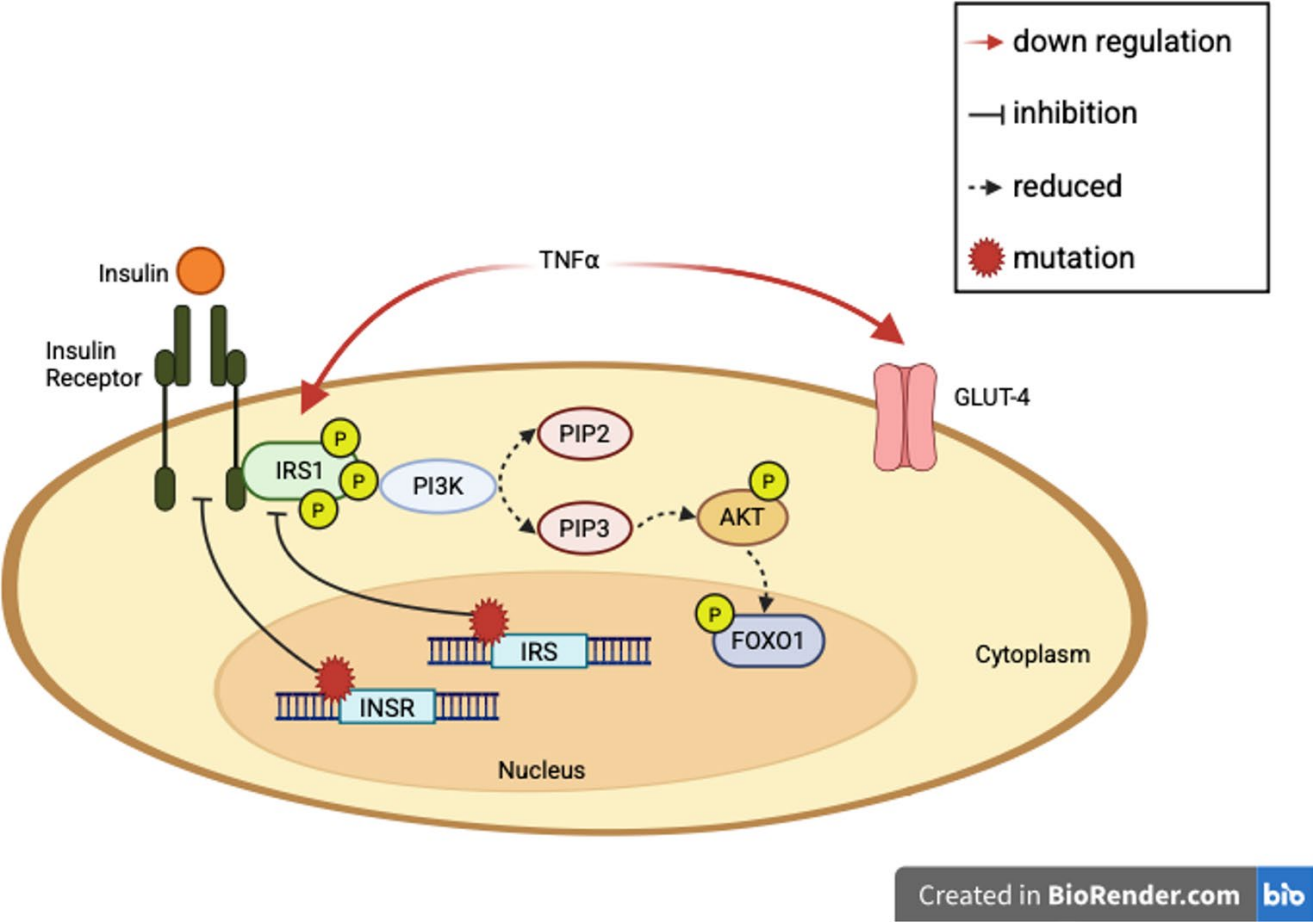
Inhibition of the phosphatidylinositol 3-Kinase-AKT pathway leading to insulin resistance. A mutation in the IRS or INSR genes can lead to decreased insulin signaling, phosphorylation of IRS1 and FOXO1. TNF⍺, an inflammatory cytokine, downregulates IRS1 and GLUT-4, causing similar effects on glucose metabolism. IRS, insulin receptor substrate; INSR, insulin receptor; PI3K, phosphatidylinositol 3-kinase; PIP, phosphatidylinositol 4,5-bisphosphate; FOXO1, forkhead box O1; GLUT-4, glucose transporter type-4

The physiological insulin response involves insulin receptors and signaling molecules, including insulin receptor substrate (IRS), phosphatidylinositol 3-kinase (PI3K), and protein kinase B (PKB or Akt) isoforms. Disturbances in these signal transduction pathways, such as a mutation in the insulin receptor (INSR) gene on chromosome 19p, can result in IR [3]. This mutation impairs insulin recognition by its receptor and limits the downstream signaling of insulin, leading to hyperinsulinemia while reducing the functional effect of insulin [3]. The cascade of phosphorylation along the PI3K-Akt pathway in the insulin response pathway is defective in IR (Fig. 1). Lack of phosphorylation of forkhead box protein O1 (FoxO1) in the Akt pathway can also lead to an imbalance in glucose metabolism [4]. Furthermore, some inflammatory cytokines, such as tumor necrosis factor-alpha (TNF- α), can inhibit the insulin signal transduction pathway by reducing the expression of IRS1 and glucose transporter type-4 (GLUT-4) [5].

In addition, modifiable lifestyle factors can contribute to IR. For example, individuals with poor dietary habits, such as not eating breakfast, have a higher risk of IR and type 2 diabetes [6]. Moreover, late and short sleep durations were associated with reduced insulin sensitivity in overweight and obese adolescents [7]. Aerobic exercises such as running and jumping a rope help improve the utilization of oxygen and its delivery in the heart and skeletal muscles. While resistance training such as lifting weights helps increase lean body mass. Both forms of exercise help reduce obesity which is a risk factor for insulin resistance [8, 9]. Exercise can also help improve insulin resistance directly by molecular mechanisms such as increasing GLUT-4 translocation to the plasma membrane [10]. The term “glucose allostasis” the fact that glucose levels remain elevated after prolonged stress, which would then lead to a higher risk of T2DM [11]. Increased stress leads to an increase in serum glucocorticoids, which counteracts the effect of insulin signaling [12].

### Inter-organ metabolic crosstalk in insulin resistance

Disturbance of inter-organ metabolic crosstalk is another important aspect of IR. Insulin signaling pathways are associated with the metabolism of glucose and lipids via the regulation of hepatic glucose production and lipolysis in white adipose tissue [13]. This was demonstrated in a study that revealed elevated hepatic acetyl coenzyme A in IR mice failed to inhibit lipolysis and hence led to hepatic glucose production [13]. The dysregulation of these processes is fundamental to IR. For example, the presence of medium-chain fatty acid metabolic markers is associated with an elevated risk of obesity-associated IR [14]. Similarly, the accumulation of triacylglycerol synthesis intermediates, such as diacylglycerol and ceramide, in adipose tissue can alter insulin sensitivity, given their roles in insulin signaling pathways in the liver and muscle tissues [15].

Hormonal imbalances have also been found to contribute to IR. For example, high levels of resistin, a hormone secreted by adipose tissue, are associated with IR. A study found that mice treated with resistin developed hyperinsulinemia as well as hyperglycemia, in which homeostasis model assessment (HOMA-IR) results suggested IR [16]. Dysregulated secretion of other adipokines, such as adiponectin and leptin, by adipose tissue and elevation of interleukin-6 can further promote IR in obese individuals [17].

### Systemic complications of insulin resistance

**Fig. 2:**
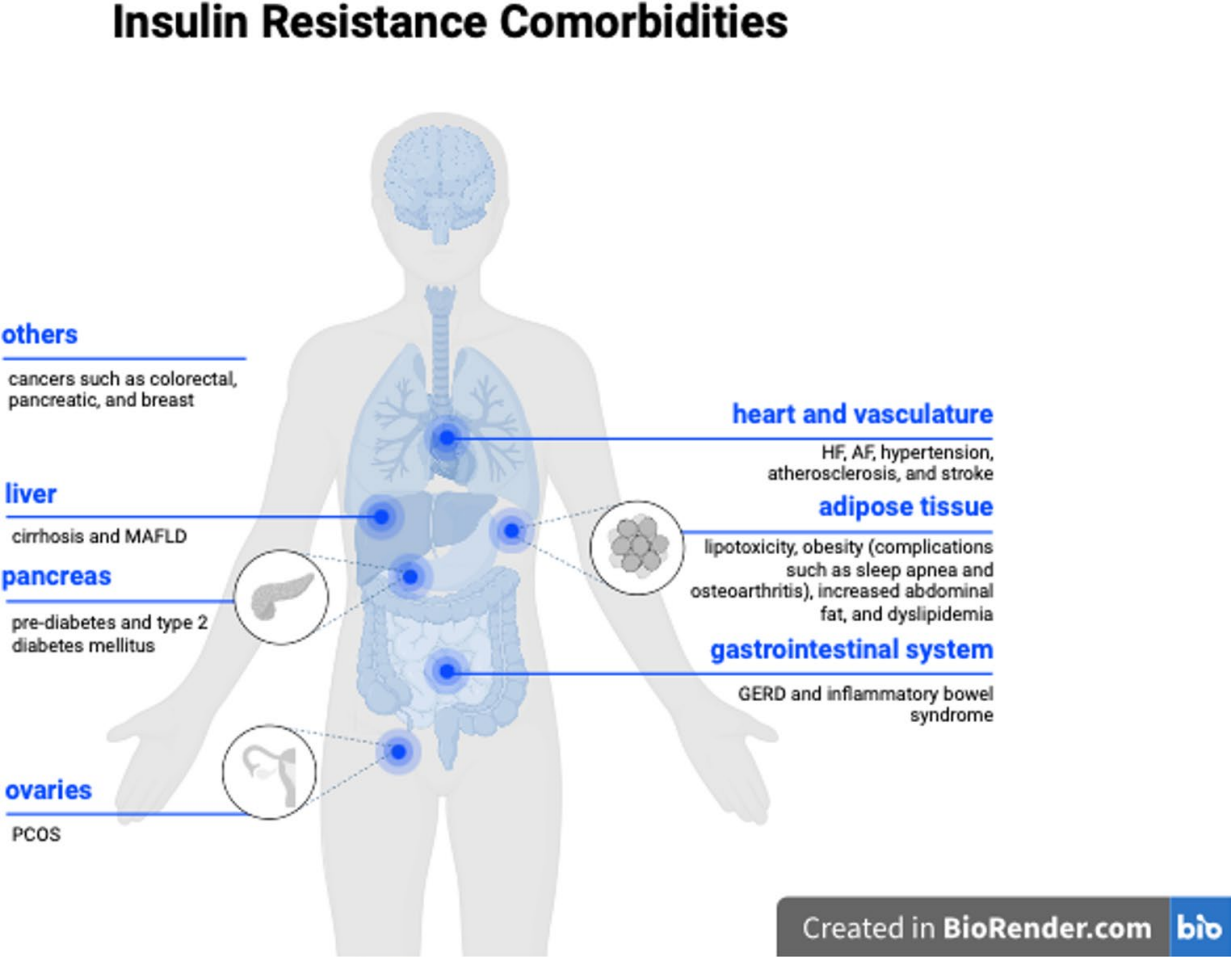
Comorbidities of insulin resistance targeted by neuromodulation. MAFLD, metabolic-associated fatty liver disease; PCOS, polycystic ovarian syndrome; HF, heart failure; AF, atrial fibrillation; GERD, gastroesophageal reflux disease

The systemic nature of IR affects organs and tissues beyond the adipose tissue, liver, and skeletal muscle, as well as other organs, including the vasculature, heart, and ovaries (Fig. 2). Persistent hyperglycemia in IR can lead to pancreatic beta cell dysfunction, which consequently causes type 2 diabetes [18]. For instance, the production of reactive oxygen species in a hyperglycemic state can induce inflammatory responses that can cause cell damage [19]. Moreover, it can also accompany other disorders, such as cardiovascular disease (CVD). For example, the synthesis of nitric oxide by the endothelium can become impaired in an insulin-resistant individual, resulting in defective vasodilation and hypertension [20]. In addition, IR is associated with reduced high-density lipoprotein and elevated triglyceride levels, which increases the risk of CVDs [21]. A previous study demonstrated that coronary artery calcium levels, a marker of atherosclerosis, are higher in individuals who are resistant to insulin [22]. Overall, changes in metabolic pathways resulting from IR can lead to hypertension, oxidative damage, and atherosclerosis, which eventually contribute to the development of CVD.

Additionally, gastrointestinal complications can occur with IR. For instance, a meta-analysis study revealed that gastroesophageal reflux disease (GERD) and diabetes mellitus are significantly associated [23]. Another study showed that diabetic patients with poor glycemic control had markedly lower distal contractile integral value and greater severity of ineffective esophageal motility when compared to those without diabetes as well as people without diabetic complications, possibly due to autonomic neuropathy affecting the esophageal function [24]. Currently, GERD is managed pharmacologically with proton pump inhibitors and histamine-2 receptor blockers, while a recent guideline also recommends surgical fundoplication in adult patients [25].

Furthermore, IR plays a significant role in the pathophysiology of metabolic-associated fatty liver disease (MAFLD), specifically in liver inflammation and cirrhosis. This stems from the role of insulin in lipid metabolism, where it promotes lipogenesis and inhibits lipolysis to reduce the fatty acid level [13]. Therefore, resistance to the anti-lipolytic action of insulin increases levels of circulating free fatty acids. The consequent increase in free fatty acids can stimulate the adipose secretory products, such as TNF-α, involved in the insulin signaling pathway [5]. Hence, modulation of TNF levels is a possible approach to improve beta cell function and treat IR [26]. In the context of hepatic insulin resistance, an increase in the rate of gluconeogenesis results in hyperglycemia [27–30]. Excess carbohydrates are converted to fatty acids in the process of de novo lipogenesis (DNL) which leads to triglyceride accumulation in the liver, also called hepatic steatosis [31–34]. This amplifies the load of fatty acid supply to the liver alongside fatty acids from dysregulated lipolysis in IR. These conditions create ER and mitochondrial stress, trigger inflammation, accumulate extracellular matrix, and eventually lead to MASH, fibrosis, and cirrhosis [35, 36]. This can also be seen indirectly as MAFLD patients are benefitting from T2DM medications like GLP-1 receptor agonists [37].

IR is associated with the development of polycystic ovarian syndrome (PCOS). Hyperinsulinemia in IR can reduce the level of sex hormone-binding globulin in circulation, which can increase the amount of free testosterone [38]. Testosterone can increase the expression of autophagy-related genes, leading to apoptosis of granulosa cells in the ovaries, which eventually leads to reduced fertility [39]. Considering the role of insulin in PCOS, a meta-analysis of 14 clinical trials showed that tackling IR by treating with a combination of metformin with glucagon-like peptide 1 (GLP-1) or thiazolidinediones improves both fasting glucose levels and hyperandrogenemia [40].

In addition, the risk of some types of cancer, including pancreatic, colorectal, and breast cancer, is higher in individuals with metabolic syndrome, also known as IR syndrome, which is a group of factors that predispose to type 2 diabetes and CVD [41]. For instance, hyperinsulinemia, a common consequence of IR, can lead to pancreatic cancer by acting directly on the insulin receptors of pancreatic acinar cells [42]. This activates the insulin receptor signaling cascade which involves PI1K/Akt/mammalian target of rapamycin (mTOR) as well as mitogen-activated protein kinase (MAPK)/extracellular signal-regulated kinase (ERK) pathways which are often mutated, leading to tumor formation [42]. In addition, IR can upregulate forkhead box protein K1 (FOXK1) expression through O-linked-β-N-acetylglucosamine transferase (OGT) mediated modification [43]. As a result, FOXK1 can dysregulate the circadian rhythm, which promotes breast cancer cell proliferation [43]. Moreover, hyperinsulinemia is associated with increased cancer mortality [44]. The correlation between IR and cancer is demonstrated through several pathways, one of which is the indirect association based on the increased risk of cancer mortality in obese individuals [45].

### Current therapeutic approaches to insulin resistance

Currently, IR is managed through lifestyle modifications and the use of insulin-sensitizing medications [46]. For instance, metformin enhances glucose transport and reduces glucose synthesis while increasing the oxidation of fatty acids and preventing lipid accumulation, contributing to beneficial effects on insulin sensitivity and cardiovascular health [47]. Moreover, metformin can also be used in the management of IR in PCOS [40]. However, metformin has several side effects, including on the gastrointestinal system, such as nausea and diarrhea. Furthermore, the greatest concern is the development of lactic acidosis that can occur in patients to which the drug is contraindicated, such as patients with hepatic dysfunction, congestive heart failure, and chronic kidney disease, so caution is advised [48].

## The nervous system in physiological control of metabolism

The nervous system plays a role complementary to that of the endocrine system and peripheral organs in metabolic regulation [49]. In particular, the autonomic division of the peripheral nervous system is the major contributor to homeostasis, and it comprises sympathetic and parasympathetic neurons. The afferent neurons of the autonomic nervous system form the ascending pathway that connects peripheral organs to the brain, while the efferent neurons form the descending pathway that conveys signals from control centers in the brain to peripheral organs. The main peripheral organs involved in metabolic control are the liver, pancreas, and adipose tissue [50]. Skeletal muscles are extensively involved in glucose regulation, not only during exercise, but also by clearing 80% of glucose following meals [51–54]. Furthermore, the influence of the nervous system on homeostasis goes beyond just regulating metabolism. It also plays a critical role in modulating the immune system response, highlighting a deep connection between these two seemingly separate functions [49].

**Fig. 3:**
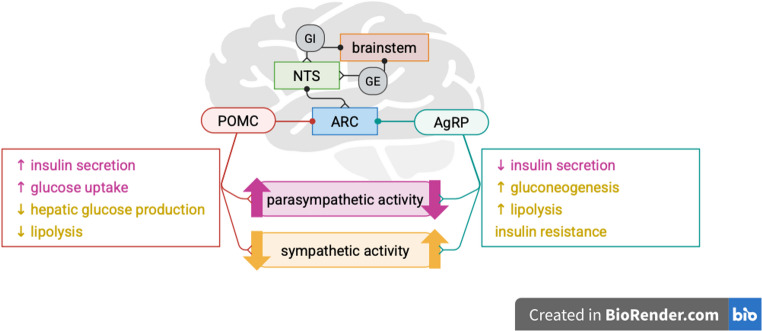
Role of hypothalamic nuclei in metabolic regulation. The NTS receive signals of metabolic states and relays them to the ARC nucleus which in turn modulates the autonomic nervous system via POMC and AgRP neurons, exerting their final effects in peripheral organs to respond to metabolic changes and restore homeostasis. NTS, nucleus tractus solitarius; GE, glucose-excited; GI, glucose-inhibited; ARC, arcuate nucleus; POMC, pro-opiomelanocortin; AgRP, agouti-related peptide

To begin with, the brain integrates signals from different tissues and organs and modulates autonomic neuronal activity to maintain metabolic homeostasis [55]. It is the site of the main autonomic control center, the hypothalamus, which contains nuclei involved in systemic metabolic regulation [56]. For instance, the arcuate nucleus of the hypothalamus (ARC) has neurons that influence appetite, namely, pro-opiomelanocortin (POMC)-expressing neurons responsible for appetite suppression, and agouti-related peptide (AgRP)-expressing neurons and tyrosine hydroxylase neurons responsible for appetite stimulation [56–60], their effects are illustrated in Fig. 3. These neurons express receptors that allow them to respond to hormones and cytokines such as leptin and insulin secreted by adipose tissue or pancreas and ghrelin secreted by the stomach to restore metabolic homeostasis [61, 62], which was shown in mice models [63, 64]. Another control center in the brain is the brainstem, which contributes significantly to energy balance. The mechanism by which the brainstem senses glucose levels allow it to detect and respond to hypoglycemia in particular. The underlying mechanism resembles that in the ventromedial nucleus of the hypothalamus. The typical neurons involved are glucose-excited (GE) and glucose-inhibited (GI) neurons. They work by adjusting the rate of signaling to respond to changes in glucose levels, which are possibly detected by other nerves and conveyed to the GE and GI via presynaptic input. The brainstem also conveys signals of metabolic states to the arcuate nucleus of the hypothalamus via the nucleus tractus solitarius (NTS) [56, 65, 66], which picks up humoral factors like glucose-like peptide 1 (GLP-1), ghrelin, and cholecystokinin (CCK) [67–71].

Apart from the brain acting as an integration center, the autonomic nervous system is the focal route by which energy state signals are conveyed. Within the autonomic system, parasympathetic nerves like the vagus nerve, innervate almost all the peripheral organs that play important roles in maintaining energy homeostasis to create a coordinated “rest-and-digest” response [50]. The vagus nerve is a vital regulator of metabolic state, particularly glycemic control, and is directly linked to the pathophysiology of diabetes and obesity [72, 73], making it a target for neuromodulation therapy in the context of metabolism as described in the next section. Its sensory fibers contain receptors for metabolic mediators, such as leptin and CCK, which allows it to relay signals about metabolic states from the peripheral tissues to the central nervous system while its efferent fibers participate in the brain-gut axis regulation of metabolism [72]. The vagal efferent innervation of islet cells in the pancreas stimulates beta cells to release insulin in response to a detected increase in glucose levels [74]. This response to sensory stimuli is called the cephalic phase of insulin release [75–77], which is reinforced by the secretion of GLP-1 upon food consumption [78–81]. Vagal innervation of the liver can also alter blood glucose levels through the inhibition of glucose production by the liver rather than increasing its uptake as [56, 82–84]. The underlying mechanism involves the effects of AgRP neurons, which are orexigenic neurons that release agouti-related peptides detected in the hypothalamus, and result in increased food-seeking behavior [60]. Hence, overexpression of AgRP is associated with obesity IR by inducing the expression of the growth factor myostatin, which decreases glucose uptake into brown adipose tissue and disrupts glucose metabolism [56, 85].

The vagal afferent and efferent nerve fibers also contribute to the coordinated regulation of the immune and metabolic systems [49]. For instance, the vagus nerve’s sensory fibers originating from the liver play a key role in monitoring the gut environment in inflammatory bowel disease (IBD) [49, 86]. This monitoring function helps maintain a healthy balance of regulatory T cells (Tregs) within the gut. Another remarkable neural circuit involving metabolic organs is the liver-brain-gut arc, where hepatic vagal afferent neurons relay signals about the microbiota of the gut to the NTS, which then conveys the signals to the gut via efferent fibers of the vagal nerve of the gut to control the differentiation of regulatory T cells [87]. In fact, different leukocytes express receptors for neurotransmitters like dopamine, acetylcholine, and norepinephrine, allowing for neural control over the suppression, activation and progression of an immune response [88–96].

**Fig. 4:**
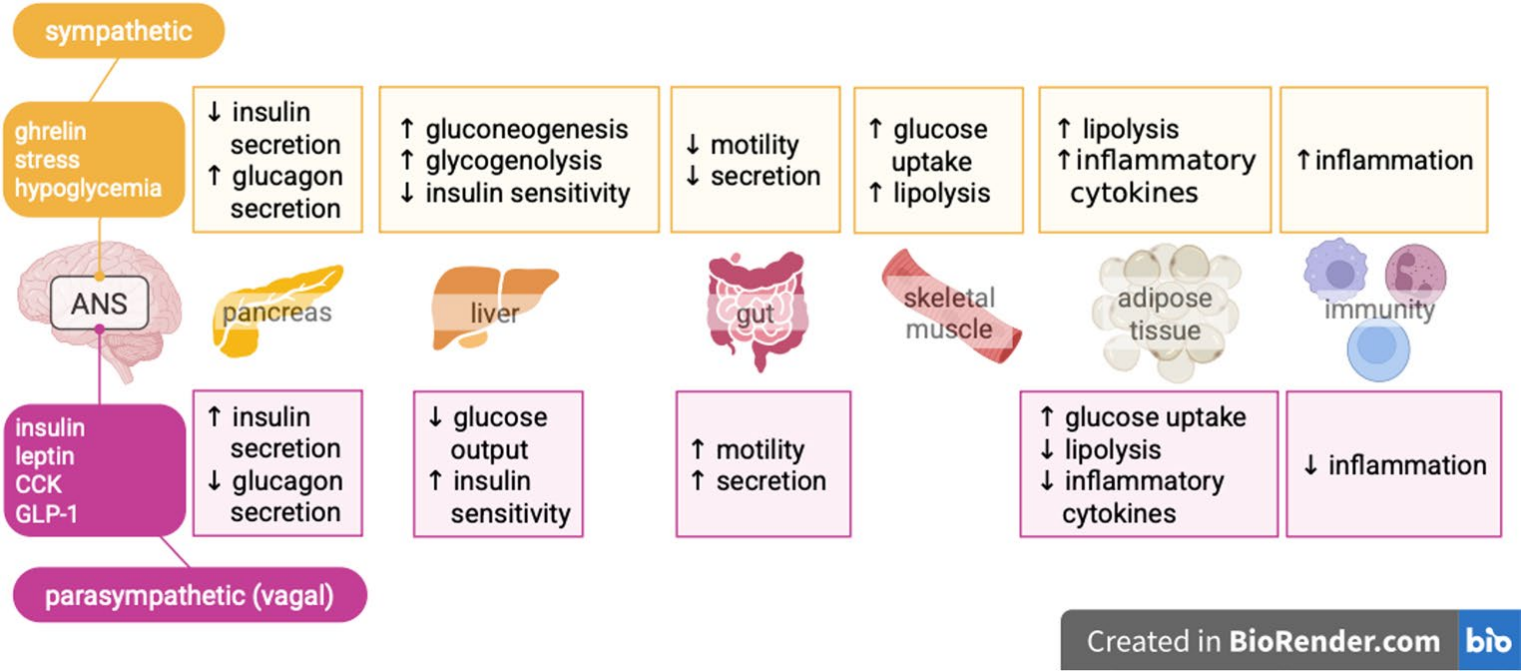
Peripheral effects of the sympathetic and parasympathetic nervous systems. Hormones released from peripheral organs are received by the autonomic nervous system (ANS) and modulate the sympathetic activity and parasympathetic activity (mainly by vagal efferents). Overactivation of the sympathetic nervous system or decreased parasympathetic activity contribute to loss of insulin sensitivity and eventually lead to peripheral insulin resistance

On the other hand, the sympathetic nervous system, the “fight-or-flight” division of the autonomic nervous system, controls energy expenditure and the rate at which metabolic processes occur. Increased sympathetic activity increases hepatic gluconeogenesis and glycogenolysis besides inhibiting hepatic glucose uptake, increasing blood glucose concentration [49, 97–100]. Over activation of the sympathetic nerves or decreased activity of the parasympathetic nerves can lead to peripheral insulin resistance [101–104]. For example, in the presence of a stressor, certain sympathetic neurons can stimulate lipolysis in white adipose tissue and gluconeogenesis in the liver and inhibit insulin production in the pancreas. In the absence of stressors, parasympathetic neurons recruit mechanisms to counteract sympathetic activity [50, 97, 98, 105–108]. The tissue-specific effects of the autonomic nervous system divisions are shown in Fig. 4.

**Fig. 5:**
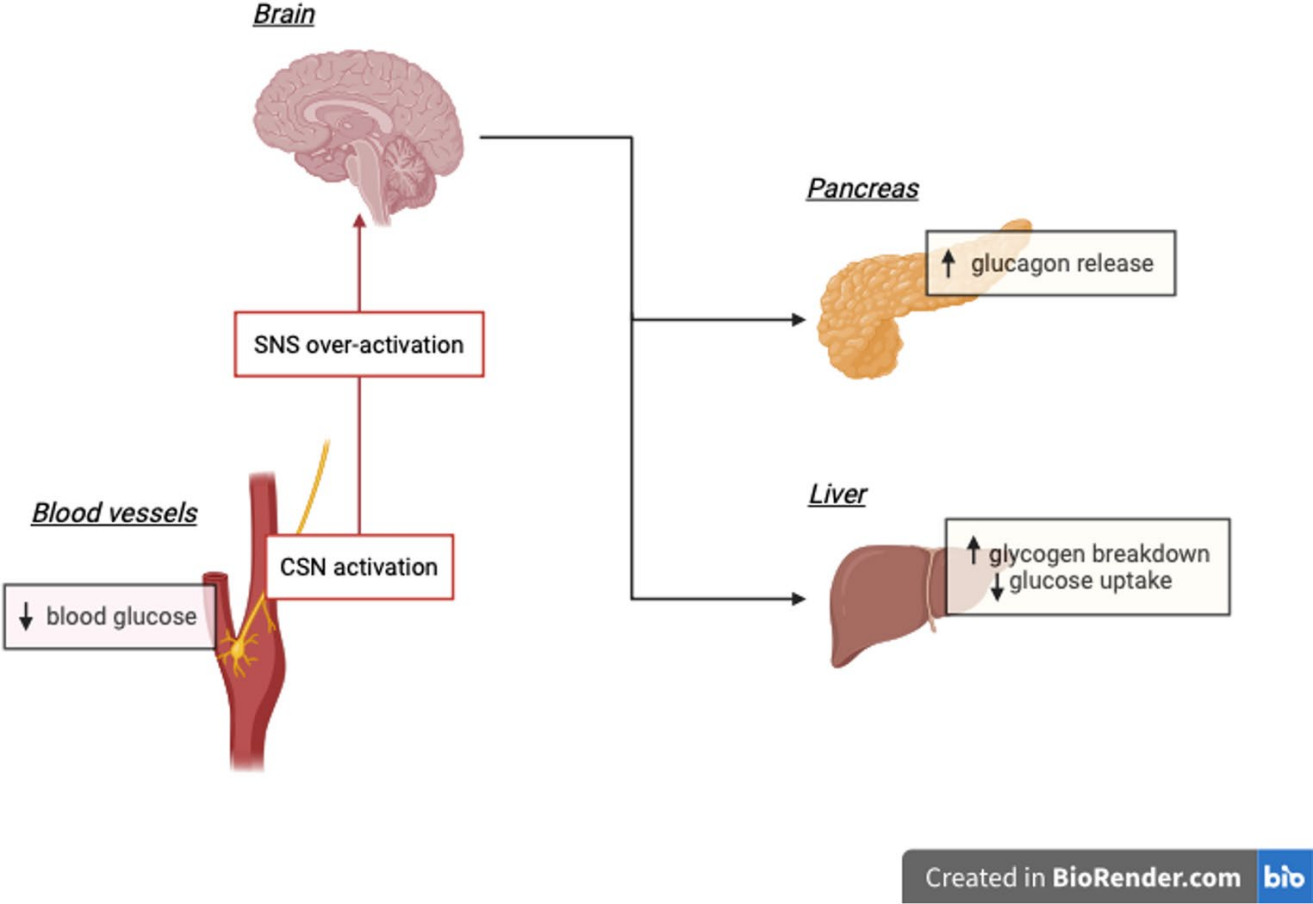
Carotid body activation of the sympathetic nervous system (SNS) via the carotid sinus nerve (CSN). Chronic sympathetic activation increases glucagon release by the pancreas, increases glycogenolysis, and decreases glucose uptake by the liver. This results in an increase in blood glucose levels, triggering persistent insulin release and overtime insulin resistance

Another contributor to glucose regulation is the carotid body, which is responsible for sensing the biochemical environment in arterial blood, as shown in Fig. 5. The role of the carotid bodies is of great importance because of their location at the bifurcation of the common carotid artery, which allows for the analysis of blood flowing to the brain [109]. When signals from the carotid body stimulate the carotid sinus nerve, they can increase the activity of the sympathetic nervous system [110, 111]. Accordingly, the carotid bodies are involved in the development of some metabolic diseases [112]. Mainly, the absence of the carotid signals prevents sympathetic over activation, hence minimizing metabolic dysregulation [113, 114]. The renal nerves also similarly contribute to glucose homeostasis. Studies, reviewed in details [101], have shown that sympathetic signaling to the renal nerve further activates the sympathetic nervous system. This positive feedback loop needs to be interrupted to prevent the loss of insulin sensitivity [101].

Understanding the neuroendocrine interaction and contributions to metabolic regulation brings in neuromodulation as a potential treatment for metabolic disorders, such as insulin resistance, or as a management plan to prevent its progression to type 2 diabetes mellitus. Neuromodulation therapy is based on the stimulation of nerves to induce a desired biological response [115].

## Bioelectronic-enabled therapy modalities with neuromodulation

### Principles of bioelectronic medicine

Bioelectronic medicine (BEM) is an emerging interdisciplinary field that uses technology to modulate the activity of the nervous system to diagnose and treat diseases. This field incorporates a diverse range of disciplines, from computer science to medicine, neuroscience, electrophysiology, signal processing, modeling, and even artificial intelligence [115, 116]. The main advantage of BEM over conventional treatments, such as pharmaceutical drugs, is its potential for personalized and autonomous treatment and reduction of side effects. In contrast, pharmacological drugs have been developed to influence specific molecular processes, resulting in a generalized effect that can lead to side effects owing to their systemic impact [117]. Unfortunately, there are diseases like rheumatoid arthritis and IBD, for which conventional pharmacological treatments have not been effective in providing a cure [115]. However, in the last years, BEM using vagus nerve stimulation (VNS) has been successful in reducing inflammatory markers in these chronic diseases, which has only been possible due to a better understanding of the vagus-mediated inflammatory reflex involved in these diseases [118].

### Techniques and applications in bioelectronic medicine for metabolic control

As described in the previous section, the contribution of the nervous system, particularly the vagus nerve, to glucose metabolism and insulin resistance increases the potential of improving the management of associated metabolic dysregulations using neuromodulation therapy (Fig. 2) [119]. For instance, the gastric pacing technique, or gastric electrical stimulation, is a technique that relies on the implantation of an electrical pacemaker in the gastric surface near the lesser curvature to stimulate the gastric vagal branches in the stomach [120]. Although the mechanisms are not fully elucidated, it has nonetheless been shown to contribute to glucose control in insulin resistance-associated obesity through a significant weight reduction, to modulate appetite in obese individuals [121, 122], and to control gastroparesis – delayed stomach-emptying of solid food – which causes bloating, distension, nausea and/or vomiting [123]. VNS has also been used to control obesity, given that recent supporting data relate obesity and diabetes to the dysregulation of autonomous nervous system (ANS) activity [124]. A study comparing weight control in rats used a spontaneous VNS device, triggered by the peristaltic activity of the stomach, to modulate food intake using electrodes directly attached to the vagal nerve for stimulation. The study found a 38% lower average weight in the VNS group compared to the control over a period of 100 days. Consistently, body fat levels showed a significant decrease in rats with implanted VNS devices [125]. Vagal blocking showed effects of appetite suppression in humans and rats, and therefore a vagal blocking device could be effective [120]. For example, the Maestro Rechargeable System has been approved by the FDA as a vagal blocker of the gastric branches for the treatment of obesity [126]. There have been also preclinical studies showing that VNS is capable of reducing blood glucose levels in rats with type 2 diabetes [127, 128]. A study of patients with depression who were treated with cervical VNS demonstrated a decrease in weight, further illustrating the use of VNS in obesity [129].

Deep brain stimulation (DBS) is a neurosurgical procedure that has traditionally been used to treat neurological disorders [130], but it has also been shown to control metabolic functions [124]. In a rat-based trial, researchers assessed the anti-obesity effects of DBS, and outcomes of reduced weight gain and suppression of appetite of diet-induced obesity (DIO) rats were observed. However, no changes were seen in chow-fed rats upon stimulation [131]. A clinical trial done on two participants displayed increased resting metabolism rates (RMR) upon electrical stimulation of the lateral hypothalamus (LH) by DBS, demonstrating its potential in the treatment of refractory – morbid obesity [132]. Another study also using DBS to stimulate LH displayed similar results of augmentation of RMR as a prospective therapy for intractable obesity. However, although mild, there were adverse effects such as nausea and anxiety [133].

### Bioelectronic medicine in insulin resistance associated comorbidities

Neuromodulation for treating the comorbidities of insulin resistance has also been a topic of interest. For instance, a recent study has investigated the effect of electrical VNS on liver inflammation and cirrhosis in mice [134]. This study demonstrated that VNS results in reduced proinflammatory molecules, such as TNFα or interleukin-1β. Moreover, the liver patatin-like phospholipase domain–containing 3 genes, required to activate hepatic stellate cells responsible for liver cirrhosis, was reduced after VNS [134]. In addition, it has been proposed that non-invasive VNS, using transcutaneous auricular VNS, may have therapeutic effects on (PCOS) by regulating metabolism, improving insulin sensitivity, and activating anti-inflammatory pathways [135].

### Bioelectronic medicine in inflammatory diseases and hypertension

Treating inflammatory comorbidities such as atherosclerosis has been another area of interest for applying bioelectronic medicine in the context of insulin resistance, motivated by the latest success of BEM for treating other inflammatory diseases like rheumatoid arthritis and IBD [118]. One part of the neural inflammatory reflex is the α7 nicotinic acetylcholine receptor subunit (α7nAChR) which has been reported to be expressed in human atherosclerotic lesions [127]. A study done on mice stimulating the nAChRα7 with the agonist GTS-21 showed a reduction in atherosclerosis in the aorta as well as having less plaque in the macrophages [88]. Therefore, VNS may be an alternative to drug-based treatment, such as the agonist GTS-21, for activating the α7nAChR or other components of the inflammatory reflex. However, a better understanding of the inflammatory reflex in atherosclerosis is needed before successful application of BEM for treating this condition [127].

### BEM in cardiovascular diseases

Neuromodulation has also been investigated for treating hypertensive patients. Cardiac neuromodulation therapy in hypertensive patients who required a dual-chamber pacemaker was found to have a significant effect on reducing average systolic blood pressure in a pilot study [128]. Treating hypertension through activation of the carotid baroreceptor has been a subject of interest for baroreflex activation therapy (BAT). The first-generation system is the Rheos system (CVRx, MN), which involves bilateral electrical field stimulation of the carotid sinus wall. Most of the clinical and experimental studies have been done using the first-generation system [136]. However, this system was associated with serious adverse effects and was substituted by the second-generation device, which is the Barostim neo system (CVRx) and it is based on unilateral carotid sinus stimulation [136]. Despite the reduced complications, the neo system resulted in less pronounced BP reductions compared with bilateral stimulation [137]. Bioelectronic medicine through interfacing with the vagus nerve has also been explored for monitoring blood pressure aiming to create a closed loop platform for blood pressure control [138]. The experiment, conducted on pigs, found that accurate blood pressure waves could be extracted from afferent left vagus signals and translated into blood pressure information [138].

VNS could also be utilized in the treatment of other cardiovascular diseases such as heart failure (HF) and atrial fibrillation (AF) [139]. As heart failure is characterized by an increase in sympathetic nervous system activity (SNS) and an abnormal decrease in the parasympathetic nervous system (PNS), restoring balance would be the key goal of neuromodulation; as such, VNS can be utilized to increase the parasympathetic tone [140, 141]. The use of VNS has been prominent in animal models, suggesting a positive effect in improving cardiac function. A study that used pacing-induced canine models demonstrated improvement in cardiac function and decreased inflammatory markers and neurohormones associated with the excitation of SNS [142]. Although human trials are limited, a study showed promising results of significant improvements in the New York Heart Association (NYHA) class, a classification system for the extent of HF, the 6-minute walk test, left ventricular ejection fraction and left ventricular volume [143]. However, it is currently accompanied by severe side effects, ranging from minor effects like cough and electrical sensations on the skin to severe effects, including death [144].

Furthermore, low-level vagus nerve stimulation (LLVNS), which is VNS delivered at lower intensities, has been implemented for the treatment of AF. The testing of LLVNS on canines showed a shortening of AF episodes coupled with an increase in the AF cycle length [145]. Furthermore, a clinical study of a small group of individuals with paroxysmal atrial fibrillation, LLVNS showed significant suppression of atrial fibrillation [146].

### Emerging techniques in bioelectronic medicine

Besides the previously mentioned VNS, a non-invasive, direct hepatic stimulation using the peripheral focused ultrasound (pFUS) showed a lower increase in abdominal fat and triglyceride levels in obese Western diet-fed mice [147]. The pFUS technique has also been investigated for the restoration of glucose homeostasis. Stimulation of the hepatoportal plexus at the porta hepatis using pFUS showed an improved level of glucose tolerance after feeding in diabetic rats [148, 149]. Neuromodulation can also affect the sphincters within the digestive tract. A clinical trial assessed the two-year efficacy and safety of a lower esophageal sphincter (LES) stimulation system that consists of an implantable pulse generator, electrical stimulation lead, and an external programmer. The results showed that LES stimulation increased the LES pressure and reduced acid reflux [150].

## Innovations and challenges in neuromodulation

The advancements presented in the previous section demonstrate the potential of neurometabolic circuits in treating metabolic diseases and offer new avenues for bioelectronic interventions. However, several challenges have to be resolved for the optimal application of neuromodulation therapies. To begin with, there is a need for a better understanding of the neural circuits and reflexes, including their role in maintaining homeostasis [115]. Advances in materials and electronics are also tackling the challenge of improving the signal-to-noise ratio (SNR) and reducing the contribution of artifacts (both physiological and stimulation-related) [115]. Innovations in this field include the use of new materials, such as a flexible 3D microelectrode array, which decreases the distance between the microelectrodes and neurons owing to the protruding nature of the 3D electrodes, thus enabling low-amplitude neural signals to be recorded [151]. Furthermore, coating the surface with materials such as conductive polymers like PEDOT ((Poly(3,4-ethylenedioxythiophene)) and PEDOT: PSS (Poly(3,4-ethylenedioxythiophene) polystyrene sulfonate), also reduces the electrical impedance, resulting in a high SNR despite the use of small electrodes [152–154].

For stimulation, one of the biggest challenges is addressing specificity to maximize the desired response, while minimizing side effects. A method using genetically guided manipulation to select specific nerve fibers can be utilized for this purpose. For example, in a study done on mice, manipulation of the expression of melanocortin-4 receptors (Mc4r) was performed, thus, allowing these fibers to be detected using a marker such as green fluorescent protein (GPF). Hence, GPF –labelled vagal fibers were identified even without histochemical processing [155, 156]. Some clinically observed side effects associated with cervical VNS are cough, hoarseness, voice alteration, and paresthesia [157–159].

Moreover, risks related to surgeries and implantation of devices, such as postoperative hematoma, infections and pain and sensory-related complications are also crucial challenges to be addressed for the successful clinical application and personalization of neuromodulation therapies [159, 160]. As such, non-invasive alternatives like VNS using ultrasound have become a topic of interest. This neuromodulation method does not require surgical placement of electrodes, which are placed on top of the skin, becoming a safer approach, and has good spatial resolution [161]. Ultrasound VNS has been researched greatly and there have been studies conducted to determine the optimal parameters, such as frequency, for it [162, 163].

Closed-loop systems, where the stimulation to be delivered adjusts according to specific physiological signals, address the issue address the issue of maintaining optimal therapeutic effects while minimizing adverse side effects by dynamically responding to the body’s real-time needs. One example is the Responsive Neurostimulator (RNS) system, which is emerging as a promising treatment option for patients with epilepsy who do not respond adequately to medication, offering real-time detection and intervention for seizure activity [164]. However, the majority of current neuromodulation models integrate the use of open-loop systems that deliver a continuous cycle of several impulses to a particular anatomic target, with the stimulation parameters predetermined clinically [165]. The traditional illustration of open and closed-loop systems is indeed found in glucose control in diabetes. Traditionally, the sensor-augmented insulin pump therapy (an open-loop system) combines continuous glucose monitoring (CGM) with insulin delivery, but patients have to manually compute the insulin dose based on CGM readings [166]. Recent studies demonstrate that advanced hybrid closed-loop systems, also known as artificial pancreas systems, over perform open-loop control [167]. For example, pediatric participants with type 1 diabetes experienced shorter hypoglycemic periods using artificial pancreas technology [140]. Artificial pancreas, require the user to only add an insulin bolus during meals and they automatically determine the basal insulin infusion rate by itself [166, 168]. One example of this hybrid closed loop is the MiniMed 780G, which is FDA approved, that uses an advanced hybrid closed loop algorithm [169, 170]. A fully closed loop would require no input from the patient and this type is currently under investigation. Given the involvement of the nervous system in the regulation of metabolic processes, it has been recently proposed to incorporate neuromodulation into the purely metabolic closed-loop systems to create a closed-loop neuromodulation system for the management of diabetes. The aim is to create a device that mimics the closed-loop system of the human body to record neural signals from peripheral nerves and metabolic biomarkers, and then use this information to adjust the neural signals in the effector and thus control glycemic fluctuations [119]. A neurometabolic closed loop system that controls insulin sensitivity by modulating the nerves and levels of insulin and glucagon through a pump has been demonstrated to be effective in silico [171].

Artificial intelligence (AI) and machine learning (ML) have also played a significant role in enabling intelligent real-time health monitoring and analysis, which aids the surge of closed-loop systems. One major hurdle in advancing the use of AI and ML algorithms is sensor performance. While these sensors provide a wealth of data, a significant portion is noise, making it difficult to extract meaningful information [172]. However, machine learning has shown promise in developing a comparative anatomical approach, which is a method of cross-species comparative analysis involving computational models. This helps overcome the challenge of integrating a huge amount of data across multiple species [173]. The integration of AI enhances and automates data collection, analysis, and security. Among the risks and ethical concerns in this field are the privacy of critical health data and the consequences of accessibility to such information [172]. The type of data offered by an AI-enhanced sensor in a closed-loop system allows for the control of biological and physical behavior raising a concern about the possibility of physical harm resulting from misuse of the data [174]. Indeed, AI applications in neuromodulation require the designing of validated and generalizable algorithms that guarantee safe therapeutic frameworks [172].

## Conclusion

We have reviewed how the brain and peripheral nervous system, particularly the vagus nerve, are involved in the regulation of processes underlying insulin resistance and comorbidities. Neuromodulation through bioelectronic medicine arises as a strong alternative to drugs for restoring faulty metabolic pathways and providing new treatment options for metabolic disorders. The future of neuromodulation in metabolic treatments lies in the continued exploration and refinement of techniques, such as VNS, for which further clinical trials and experimentation on animal models are required. In addition to the challenges presented in the previous section, which are shared for all neuromodulation applications, the complex multisystem nature of insulin resistance increases the complexity of the problem. This complexity has been highlighted broadly in this paper, encompassing areas such as its effects on the reproductive, gastrointestinal, cardiovascular, and endocrine systems- within the shell of various diseases beyond diabetes mellitus. Therefore, it is essential to understand the basic mechanisms underlying the neuro-metabolic reflex in insulin resistance to create safer and more successful solutions.

## Data Availability

No datasets were generated or analysed during the current study.
