# Correction to: Neuromodulation using Bioelectronics-enabled therapies to combat insulin resistance and its comorbidities

**DOI:** 10.1007/s40200-026-01932-5

**Published:** 2026-03-05

**Authors:** Yosra Magdi Mekki, Jeongeun Park, Zeinah Alzaareer, Zaeem Hussain, Ayesha Gulzar, Abdelnaser Elzouki, Amparo Güemes, Susu M. Zughaier

**Affiliations:** 1 Department of Basic Medical Sciences, College of Medicine, QU Health, Qatar University, Doha, Qatar; 2 Department of Engineering, Electrical Engineering Division, University of Cambridge, 9 JJ Thomson Ave, Cambridge, CB3 0FA UK; 3 Department of Internal Medicine, Hamad Medical Corporation, Doha, Qatar; 4 Weill Cornell College of Medicine, New York, NY USA; 5 Weill Cornell College of Medicine, Doha, Qatar

**Correction to: Journal of Diabetes & Metabolic Disorders (2026) 25:59**

10.1007/s40200-026-01876-w

In this article the Author contributions statement was incorrectly given as ‘A.G.G. provided scientific mentorship, guidance and feedback’. but should have been ‘A.Gü. provided scientific mentorship, guidance and feedback’.

Incorrect version:

A.G.G. provided scientific mentorship, guidance and feedback .

Corrected version:

A.Gü. provided scientific mentorship, guidance and feedback.

The original article has been corrected.

